# FACTORS INFLUENCING AMERICAN ELDERS’ FORMAL CIVIC ENGAGEMENT: A SYSTEMATIC LITERATURE REVIEW

**DOI:** 10.1093/geroni/igz038.2397

**Published:** 2019-11-08

**Authors:** Haiping Chen, Fengzhi Ma

**Affiliations:** Department of Sociology, Peking University, Beijing, China

## Abstract

Summary: Today’s American elders are increasingly being seen as an asset to society through civic engagement. A new challenge facing America now and in the years ahead is how to tap such asset. Formal organizations should play a leading role in institutionalizing American elders’ civic engagement. Due to a structural lag between social changes and organizational practices, however, many organizations are not ready to engage the large number of American elders. To address this issue, formal organizations first need to know which factors affect American elders’ civic engagement and then are able to come up with effective solutions. Although some studies have investigated the contributing factors of American elders’ civic engagement, there are very few systematic syntheses of these factors from various different studies. To fill this gap, the authors conducted a mixed methods systematic literature review using meta-summary. Through electronic search of five databases and hand search of bibliographies, 22 articles were used in final analysis based on eligibility criteria, including 19 quantitative studies, two qualitative studies and one mixed methods study. Findings: The review identified six themes and 28 factors related to American elders’ civic engagement. These themes encompassed socio-demographic factors (eight factors), health status (four factors), program characteristics (four factors), engagement opportunities (three factors), engagement outcomes (five factors) and social capital (four factors). Applications: Formal organizations are advised to develop relevant competencies to capture the influences of identified factors. Social workers are also required to develop multilevel competencies to better engage American elders with the organizational settings.

